# The histone methyltransferase EZH2 as a novel prosurvival factor in clinically aggressive chronic lymphocytic leukemia

**DOI:** 10.18632/oncotarget.9371

**Published:** 2016-05-14

**Authors:** Nikos Papakonstantinou, Stavroula Ntoufa, Elisavet Chartomatsidou, Konstantia Kotta, Andreas Agathangelidis, Lefki Giassafaki, Tzeni Karamanli, Panagiota Bele, Theodoros Moysiadis, Panagiotis Baliakas, Lesley Ann Sutton, Niki Stavroyianni, Achilles Anagnostopoulos, Antonios M. Makris, Paolo Ghia, Richard Rosenquist, Kostas Stamatopoulos

**Affiliations:** ^1^ Institute of Applied Biosciences, Center for Research and Technology Hellas, Thessaloniki, Greece; ^2^ Department of Immunology, Genetics and Pathology, Science for Life Laboratory, Uppsala University, Uppsala, Sweden; ^3^ Division of Experimental Oncology and Department of Onco-Hematology, IRCCS San Raffaele Scientific Institute and Università Vita-Salute San Raffaele, Milan, Italy; ^4^ Hematology Department and HCT Unit, G. Papanicolaou Hospital, Thessaloniki, Greece

**Keywords:** CLL, EZH2, apoptosis, proliferation, inhibitors

## Abstract

The histone methyltransferase EZH2 induces gene repression through trimethylation of histone H3 at lysine 27 (H3K27me3). EZH2 overexpression has been reported in many types of cancer and associated with poor prognosis. Here we investigated the expression and functionality of EZH2 in chronic lymphocytic leukemia (CLL). Aggressive cases with unmutated IGHV genes (U-CLL) displayed significantly higher EZH2 expression compared to indolent CLL cases with mutated IGHV genes (M-CLL); furthermore, in U-CLL EZH2 expression was upregulated with disease progression. Within U-CLL, EZH2^high^ cases harbored significantly fewer (*p* = 0.033) *TP53* gene abnormalities compared to EZH2^low^ cases. EZH2^high^ cases displayed high H3K27me3 levels and increased viability suggesting that EZH2 is functional and likely confers a survival advantage to CLL cells. This argument was further supported by siRNA-mediated downmodulation of EZH2 which resulted in increased apoptosis. Notably, at the intraclonal level, cell proliferation was significantly associated with EZH2 expression. Treatment of primary CLL cells with EZH2 inhibitors induced downregulation of H3K27me3 levels leading to increased cell apoptosis. In conclusion, EZH2 is overexpressed in adverse-prognosis CLL and associated with increased cell survival and proliferation. Pharmacologic inhibition of EZH2 catalytic activity promotes apoptosis, highlighting EZH2 as a novel potential therapeutic target for specific subgroups of patients with CLL.

## INTRODUCTION

Aberrations in chromatin-modulation mechanisms play a central role in cancer initiation and progression. [1, 2] The histone methyltransferase (HMT) Enhancer of Zeste Homolog 2 (EZH2) is the catalytic core protein in the Polycomb Repressor Complex 2 (PRC2). EZH2 catalyses the trimethylation of histone 3 lysine 27 (H3K27me3) and mediates the silencing of target genes involved in fundamental cellular processes, such as cell fate decision, cell cycle regulation, cell differentiation, senescence [3] and cancer. [4] Regarding the latter, EZH2 is overexpressed in several cancer types, both solid tumors and hematopoietic malignancies [4, 5] and its overexpression is correlated with disease aggressiveness. [6–9]

EZH2 is critically implicated in embryonic development [10] and also participates in maintaining the multi-potency of adult stem cells in various contexts, including the long-term self-renewal potential of hematopoietic stem cells by switching off pro-differentiation genes and promoting a shift toward proliferation by increasing expression of genes which facilitate progression through the cell cycle. [11] Furthermore, EZH2 plays a key role in B cell ontogeny, where it is highly expressed in lymphoid progenitors [12], declines in resting B cells, but is then massively up-regulated when activated B cells form germinal centers (GCs), wherein they undergo rapid proliferation and immunoglobulin affinity maturation. [13] These observations suggest an important role for EZH2 in GC B-cell proliferation and a possible contribution to the pathogenesis of lymphomas derived from GC B cells. This claim is supported by the discovery of activating mutations in the EZH2 SET domain in a proportion of both diffuse large B cell lymphoma of the GC subtype (GC-DLBCL) and follicular lymphoma (FL). [14]

A recent study [15] from our group implicated for the first time EZH2 in the pathophysiology of chronic lymphocytic leukemia (CLL), a chronic malignancy of mature B cells [16], displaying remarkable clinical and biological heterogeneity. In more detail, we found that EZH2 was highly expressed in a subgroup of patients (subset #1) [15], characterized by a stereotyped B cell receptor (BcR) with unmutated immunoglobulin heavy variable genes (U-CLL) and an aggressive disease course [17, 18]. Moreover, we reported that EZH2 expression is modulated by miR-101, a well known epi-miRNA, which was downregulated in CLL subset #1 [15] since changes in miR-101 expression brought about by silencing using miR-101 inhibitor or overexpression using miR-101 mimic affected EZH2 protein expression in primary CLL cells [15].

Prompted by these preliminary observations, here we sought to investigate in more detail EZH2 expression patterns and functionality in CLL. We report significantly higher EZH2 mRNA and protein expression levels in adverse-prognosis U-CLL cases compared to more indolent cases with mutated immunoglobulin heavy variable genes (M-CLL). We also show that EZH2 expression is associated with CLL cell proliferation as well as resistance to apoptosis, while downregulation of EZH2 expression or pharmacological inhibition of EZH2 catalytic activity leads to apoptosis, highlighting a crucial role for EZH2 in CLL cell homeostasis. On these grounds, EZH2 emerges as a novel potential therapeutic target, at least for specific subgroups of patients with CLL.

## RESULTS

### EZH2 is overexpressed in U-CLL and its expression is associated with miR-101

We analyzed EZH2 mRNA expression in a cohort of 131 CLL cases of whom 75 (57.3%) and 56 (42.7%) concerned M-CLL and U-CLL, respectively. Following established criteria for stereotyped subset assignment [19, 20], 46/131 (35%) cases were classified to the major stereotyped subsets namely subset #1 (U-CLL-aggressive; *n* = 9), #2 (mixed M-CLL and U-CLL, aggressive; *n* = 11), #4 (M-CLL, indolent; *n* = 11), #6 (U-CLL, aggressive; *n* = 7), #8 (U-CLL, aggressive, *n* = 7) (Supplemental Table 1) [21, 22].

**Figure 1:**
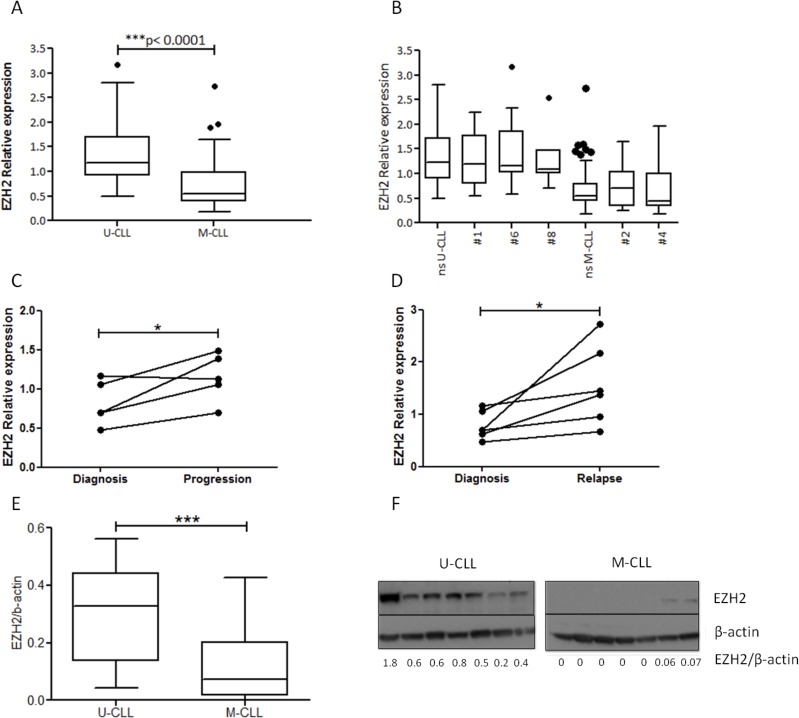
EZH2 is overexpressed in U-CLL at both the mRNA and protein level **A.** U-CLL cases (*n* = 56) express higher EZH2 mRNA levels compared to M-CLL cases (*n* = 75) (FD>2). The Tukey whisker plots show EZH2 relative expression. **B.** Comparison of the cases belonging to major stereotyped subsets versus non-subset (ns) M-CLL and U-CLL cases. The Tukey whisker plots show EZH2 relative expression. **C.**-**D.** Analysis of serial samples obtained over a period spanning 2-7 years. In the graph two connected points represent EZH2 relative expression in the two different time points (C) diagnosis versus progression (*n* = 5) (D) diagnosis *vs* relapse (*n* = 6) **E.**-**F.** Significantly higher EZH2 protein levels in U-CLL versus M-CLL. (E) The Tukey whisker plots show EZH2 protein levels normalized to β-actin (F) western blotting for EZH2 protein levels for 7 M-CLL and & U-CLL cases. *p < 0.05, ***p < 0.0001.

U-CLL cases were found to express significantly higher EZH2 mRNA levels compared to M-CLL cases (fold difference, FD>2, *p* < 0.00001) (Figure 1A). No differences were identified regarding EZH2 expression levels between subset versus non-subset cases or different subsets with similar SHM status (Figure 1B). In contrast, significant differences emerged between stereotyped subsets with different SHM status (Supplemental Table 2). Of note, EZH2 levels were low in clinically aggressive subset #2, thus sharply contrasting aggressive, U-CLL stereotyped subsets #1, #6 and #8 (Figure 1B). On these grounds, we conclude that EZH2 mRNA levels are higher in U-CLL, independently of BcR IG stereotypy.

Analysis of serial samples obtained over a period spanning 2-7 years from 6 progressive U-CLL cases (Supplemental Table 3), revealed that EZH2 mRNA levels significantly increased at disease progression (FD = 1.6, *p* < 0.05) and relapse (FD = 2, *p* < 0.05) compared to diagnosis, consistent with the notion that disease aggressiveness is correlated with high EZH2 levels (Figure 1C, 1D). The results were confirmed also at protein levels using western bloting (Supplemental Figure 1A).

EZH2 protein expression analysis revealed similar results, in that significantly higher (*p* < 0.0001) expression levels were found in U-CLL (*n* = 20) versus M-CLL (*n* = 25) (Figure 1E, 1F). Moreover, EZH2 mRNA levels correlated significantly (*r* = 0.4, *p* < 0.005) with EZH2 protein levels (Supplemental Figure 1B).

We previously reported that miR-101 regulates EZH2 expression in aggressive stereotyped CLL subset #1, showing significant anti-correlation with EZH2 expression levels. [15] Here we extended our microRNA profiling analysis to an additional 16 U-CLL and 22 M-CLL and confirmed a significant (*r* = −0.6, *p* < 0.005) inverse correlation between EZH2 mRNA levels and miR-101 levels in U-CLL where EZH2 levels are high (Supplemental Figure 2A, B). These results highlight miR-101 as a modulator of EZH2 expression in U-CLL in general.

### In CLL the expression of PRC2 components correlates to the expression of EZH2

**Figure 2:**
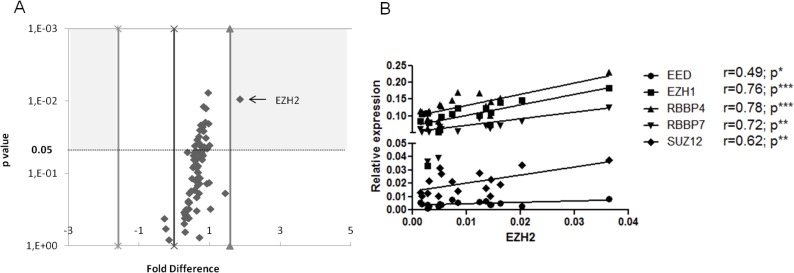
Expression analysis of Polycomb and Trithorax group genes in CLL **A.** Volcano plot depicting the differential expression of the 84 molecules analyzed in 9 U-CLL *vs* 8 M-CLL cases. The y axis depicts the p value in logarithmic scale and the x axis the fold difference. **B.** Correlation of EZH2 levels with the levels of other PRC2 components. The x axis represents EZH2 relative expression while the y axis represents EZH1, EED, RBBP4, RBBP7 and SUZ12 relative expression. For each gene, there is a statistically significant positive correlation with EZH2 levels (*r* = 0.49-0.78). *p < 0.05, **p < 0.001, ***p < 0.0001.

Other polycomb group (PcG) proteins besides EZH2 have oncogenic potential [3], while proteins counteracting PcG function e.g. the Trithorax group (TrxG) proteins are often implicated in cancer. [23] With this in mind, we used PCR arrays to analyze the mRNA expression of 86 genes associated with the PcG or TrxG complexes, including chromatin modification enzymes and remodeling factors, in 9 U-CLL and 8 M-CLL cases. Almost all genes were overexpressed in U-CLL cells compared to M-CLL, though not reaching statistical significance with the exception of EZH2, (Supplemental Table 4; Figure 2A). Focusing on the main PRC2 components, namely SUZ12, EED, EZH1 and RBBP4/7, their mRNA levels were significantly correlated (*r* = 0.49-0.78; *p* < 0.05) with EZH2 mRNA levels (Figure 2B).

### EZH2 expression induces H3K27me3 trimethylation and confers CLL cells a survival advantage

The comparison of EZH2 mRNA levels between U-CLL and M-CLL (Figure 1A) showed that U-CLL generally expressed higher EZH2 levels, yet there were outliers, while occasional M-CLL cases showed high EZH2 expression levels. For this reason, in order to explore the functional impact of differential EZH2 expression in CLL, cases were classified into EZH2^high^ and EZH2^low^ subgroups based on EZH2 mRNA levels using ROC curve and Youden index statistical tests.

**Figure 3:**
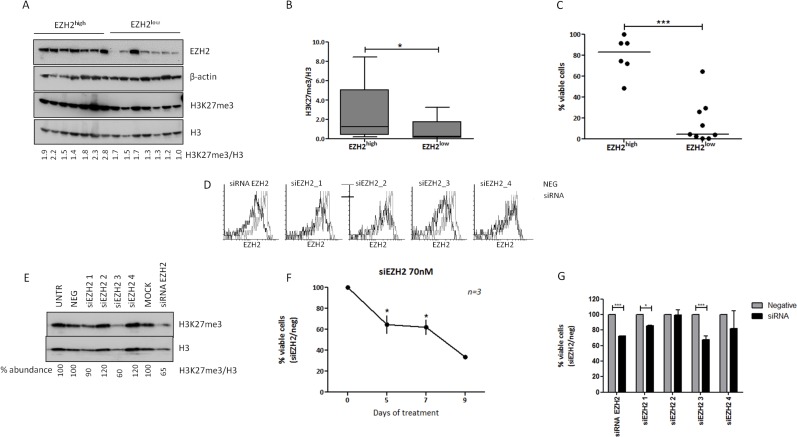
EZH2 expression induces trimethylation of H3K27 in CLL cells, while EZH2 downregulation with siRNA leads to apoptosis Analysis of H3K27me3 using western blotting in **A.** 7 EZH2^high^ and 7 EZH2^low^ representative cases and **B.** all cases analyzed (19 EZH2^high^ versus 34 EZH2^low^ cases). The Tukey whisker plots depict the levels of H3K27me3 normalized to histone H3 levels. **C.** CLL cell viability analysis using annexin V/PI staining in CD19^+^ CLL cells after 9 days in culture for 6 EZH2^high^ and 9 EZH2^low^ cases. The x axis shows the percentage of viable cells normalized to day 0. The black line corresponds to the median value. **D.** Levels of EZH2 (black lines) expression, in one representative case, as measured by flow cytometry after transfection with the negative control (grey lines) or siRNAs against EZH2. siRNA-EZH2 refers to the initial siRNA used for all experiments. The other siRNAs represent the four clones that used for validation **E.** H3K27me3 levels after EZH2 knock-down using different siRNAs in one representative case. **F.** Cell viability analysis at different time points after treatment of primary cells with siRNA for EZH2. The x axis shows the percentage of viable cells normalized to the negative control. **G.** Cell viability analysis at day 5, after treatment of primary cells with all siRNAs used in the study for EZH2. The x axis shows the percentage of viable cells normalized to the negative control *p < 0.05, ***p < 0.0001.

First, we analyzed the levels of the EZH2 mark H3K27me3 using Western blotting. We found that EZH2 protein levels and levels are positively correlated (*r* = 0.5, *p* < 0.001) (Supplemental Figure 3). Moreover, EZH2^high^ cases (*n* = 19) displayed higher H3K27me3 levels compared to EZH2^low^ cases (*n* = 34) (*p* < 0.05) (Figure 3A, 3B).

Next, in order to study the effect of EZH2 expression on CLL cell viability, we studied CLL cell apoptosis after 9 days in culture in 6 EZH2^high^ and 9 EZH2^low^ cases. We found that EZH2 levels correlated with cell viability, since cells from EZH2^high^ cases displayed significantly higher viability (*p* < 0.0001) compared to EZH2^low^ cases (Figure 3C).

In order to explore whether the difference in cell viability could be attributed to EZH2 levels, we tampered with EZH2 expression in CLL cells from 3 EZH2^high^ cases using an EZH2-specific siRNA and observed significant downregulation of EZH2 followed by a more modest downregulation of H3K27me3 levels (Figure 3D, 3E). Cell viability analysis revealed a time-dependent increase in cell apoptosis, indicating that EZH2 expression confers a survival advantage to CLL cells (Figure 3F). In order to validate our results we also performed the same experiments using additional 4 EZH2-specific siRNAs with different sequences. Two out of four siRNAs reduced EZH2 and H3K27me3 levels (Figure 3D, 3E) and CLL cell viability at day 5 (Figure 3G), confirming our results.

**Figure 4:**
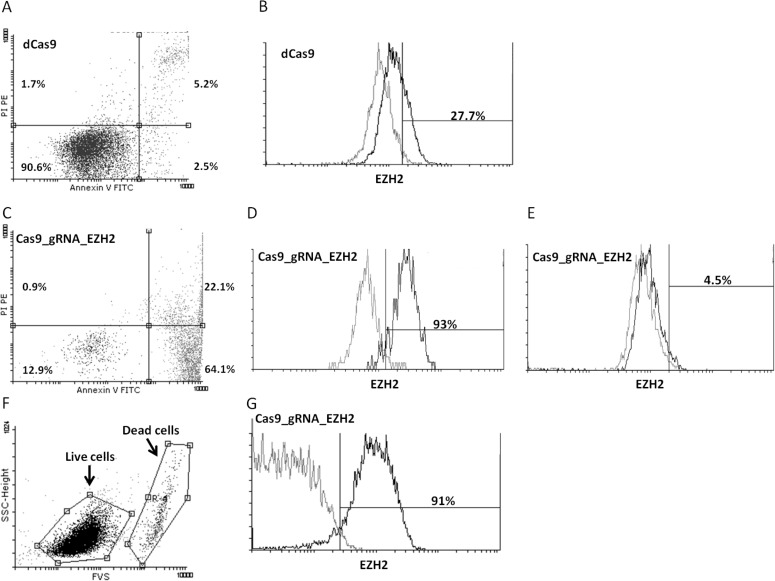
Downregulation of EZH2 expression in MEC1 CLL cells by the CRISPR/Cas9 approach leads to apoptosis Dot-plot depicting cell viability as measured by Annexin V/PI staining in the MEC1 CLL cell line transduced with **A.** lentiviral constructs which express an inactive Cas9 as control (dCas9) or **C.** co-express active Cas9 together with an EZH2 target guide RNA (gRNA). Histograms depicting EZH2 expression levels compared to isotypic control in **B.** the dCas9 MEC1 cell line and the Cas9_gRNA_EZH2 MEC1 cell line gated on **D.** live cells (Annexin V-/PI-) and **E.** and apoptotic (Annexin V^+^/PI^−^) cells. **F.** 35 days post transfection viability was measures using the FVS viability dye and the great majority of the cells were alive and (G) EZH2 possitive.

Additional evidence about the importance of EZH2 expression for CLL cell viability was obtained with an alternative genome editing approach. In more detail, we transduced the MEC1 CLL cell line with lentiviral constructs which either co-express active Cas9 with an EZH2 target guide RNA (gRNA) (Cas9_gRNA_EZH2), or an inactive Cas9 as control (dCas9). Twenty days after transduction and selection, control cells exhibited 90.6% viability (Figure 4A); the percentage of EZH2-positive control cells was 27.7% (Figure 4B). In contrast, cells expressing active Cas9 targeting EZH2 showed a dramatic decrease in viability with only 13% viable cells (Figure 4C). Strikingly, the great majority (93%) of surviving cells were EZH2-positive (Figure 4D), while the apoptotic cells (Annexin V^+^/PI^−^) were EZH2-negative (Figure 4E). Finally, 35 days after transduction, control cells were in the same condition while cells expressing active Cas9 targeting EZH2 showed a dramatic increase in cell viability (93%), probably due to the remaining EZH2 positive clone, and as expected the majority of the cells were EZH2 positive (91%) (Figure 4F, 4G).

### Proliferating CLL cells express EZH2

In order to investigate if EZH2 is linked to CLL cell proliferation, using flow cytometry we assessed EZH2 and Ki67 expression in primary CLL cells and the MEC1 CLL cell line. First, we noted that EZH2 expression was restricted to a fraction of the CLL clone ranging from 11-35% (Supplemental Figure 4A, 4B). Interestingly, the EZH2 positive compared to the negative fraction showed higher expression of CD38 (Fold difference = 1.6, *p* < 0.0005), and higher mean fluorescence intense (MFI) of CD5 (Fold difference = 1.25, *p* < 0.005), both markers of cell activation (Supplemental Figure 4C-F).

**Figure 5:**
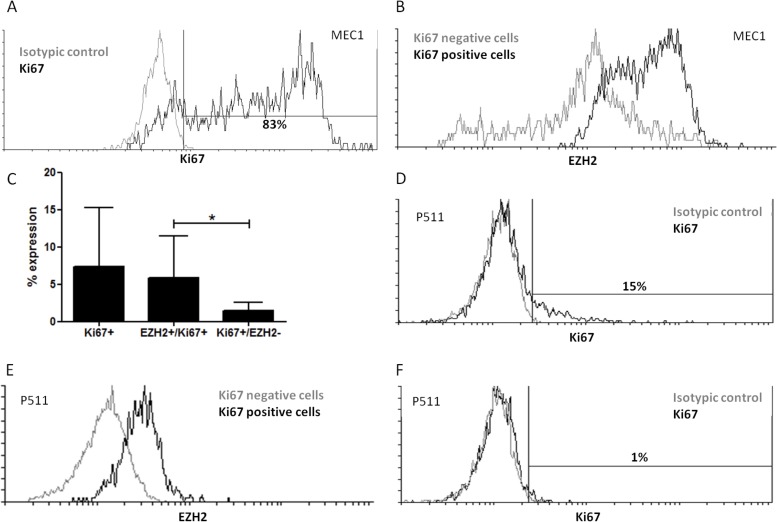
Proliferating primary and MEC1 CLL cells express EZH2 **A.** Analysis of CLL cell proliferation using Ki67 staining in the MEC1 CLL cell line. Overlay histogram for Ki67 expression in MEC1 cells (black histogram); grey histogram: isotypic control. **B.** Cells were gated on Ki67^+^ cells and Ki67^−^ fractions according to the isotypic control and EZH2 expression was measured in the two populations. The overlay histogram shows the EZH2 expression in Ki67^+^ cells (black histogram) and Ki67^−^ cells (grey histogram). **C.** Ki67 expression in primary CLL cells from 5 cases. Most Ki67^+^ cells co-express EZH2 (EZH2^+^/Ki67^+^). The y axis shows the percentage of positive cells for Ki67 expression; *p < 0.05. **D.** Overlay histogram for Ki67 expression in primary CLL cells (black histogram) for one representative EZH2^high^ case; grey histogram: isotypic control. **E.** Cells were gated on Ki67^+^ cells and Ki67^−^ fractions according to the isotypic control and EZH2 expression was measured in the two populations, for one representative CLL case. The overlay histogram shows the EZH2 expression in Ki67^+^ cells (black histogram) and Ki67^−^ cells (grey histogram). **F.** Overlay histogram for Ki67 expression in primary CLL cells (black histogram) for one representative EZH2^low^ case; grey histogram: isotypic control.

MEC1 CLL cells show persistent proliferation with a doubling time of 40 hours. Our analysis showed that 83% of the cells were Ki67^+^ (Figure 5A); of these 84.3% co-expressed EZH2 (Figure 5B). Although peripheral blood primary CLL cells showed lower Ki67 levels than MEC1 cells (range of Ki67^+^ cells: 2%-20%) (Figure 5C, 5D), in EZH2^high^ cases most Ki67^+^ cells co-expressed EZH2 (Figure 5C, 5E): in more detail, in the four EZH2^high^ cases analysed, 75-97% of Ki67^+^ cells were also EZH2^+^. As a control we performed the same analysis for two EZH2^low^ cases, with a different pattern emerged depending on SHM status: in particular, the M-CLL/EZH2^low^ case analyzed was negative for Ki67^+^ cells (Figure 5F), while in the U-CLL/EZH2^low^ case, though few Ki67^+^ cells were detected, 62% of these co-expressed EZH2.

### Pharmacological inhibition of EZH2 catalytic activity promotes CLL cell apoptosis

**Figure 6:**
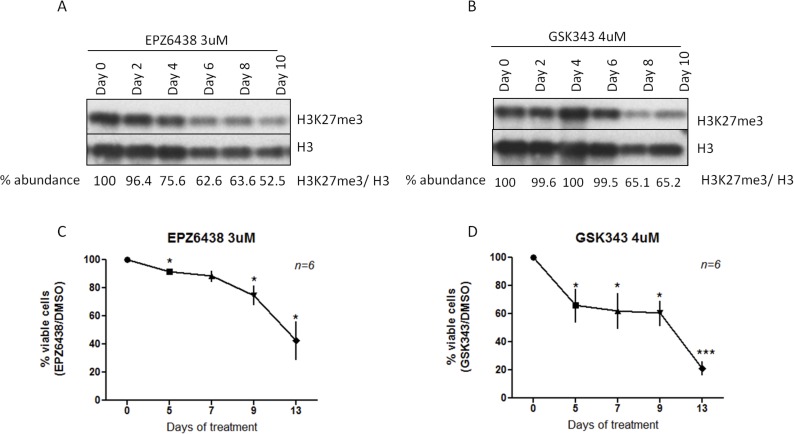
Pharmacological inhibition of EZH2 catalytic activity **A.** Western blotting analysis of H3K27me3 levels in one representative case after treatment with EPZ6438 for different time points. **B.** Western blotting analysis of H3K27me3 levels in one representative case after treatment with GSK343 for different time points. **C.** and **D.** Effects of incubation with EPZ6438 and GSK343 for 13 days on CLL cell viability as measured by annexin V/PI stining using flow cytometry. For both graphs, the x axis shows the percentage of viable cells normalized to control cells that were treated with DMSO. *p < 0.05, ***p < 0.0001.

Prompted by these findings, we sought to obtain more supportive evidence for the anti-apoptotic role of EZH2 in CLL using two EZH2 pharmacological inhibitors, namely GSK343 and EPZ6438. First, in order to determine the effective concentrations of these inhibitors, we treated 5 EZH2^high^ cases with different concentrations of GSK343 and EPZ6438, and measured CLL cell viability at different time points (days 5, 7, 9, 13), which resulted in concentration-dependent reductions in CLL cell viability (Supplemental Figure 5A-B). Moreover, in three EZH2^high^ cases we treated cells with different concentrations of GSK343 and EPZ6438, which induced concentration-dependent reduction in global levels (Supplemental Figure 5C, 5D). Based on this analysis, we chosen the first concentration which reduced H3K27me3 levels and induced statistically significant difference in CLL cell viability. For EPZ6438 we chose 3uM as the effective dose and 6 additional EZH2^high^ cases were treated for a total of 10 days resulting in a time-dependent reduction of H3K27me3 levels starting from day 2 (Figure 6A). Similarly, treatment of the same cases with 4uM GSK343 induced reduction of H3K27me3 levels, albeit at later time points (starting from day 6; Figure 6B). We also assessed the effects of these inhibitors on cell apoptosis after incubation of the same 6 EZH2^high^ cases with EPZ6438 (3 uM) and GSK343 (4uM) for 13 days and found decreased cell viability after 5 days of exposure to either inhibitor compared to the control cells (DMSO-treated) (Figure 6C and 6D). Both inhibitors decreased CLL cell viability in a time-dependent manner, suggesting that the histone trimethylation catalytic activity of EZH2 is vital for CLL cell survival. Interestingly, the same treatment of 3 EZH2^low^ cases revealed that viability of EZH2^high^ cases are more dependent on EZH2 function than EZH2^low^ cases (Supplemental Figure 6A, 6B).

### Clinicobiological correlations

As already mentioned, EZH2 expression was strongly associated with IGHV gene SHM status, being significantly higher in U-CLL (*p* < 0.00001). Interestingly, amongst U-CLL, EZH2 expression was homogeneous in cases lacking any SHM (100% IGHV gene identity to the germline) and those with borderline SHM status (98-99.9% identity). Regarding the remaining clinicobiological features, high EZH2 expression was associated with advanced clinical stage and high ZAP70 expression (*p* = 0.003 and *p* = 0.01 respectively, Supplemental Table 5). Notably, however, an inverse correlation was noted between EZH2 expression levels and *TP53* gene abnormalities (*TP53*abn: del(17p) and/or *TP53* mutations) amongst U-CLL, in that U-CLL/EZH2^high^ cases harbored significantly fewer (*p* = 0.033) *TP53*abn (2/27 cases, 7%) compared to U-CLL/EZH2^low^ cases (6/19, 32%). No association between EZH2 expression and other genomic lesions known to be recurrent in CLL was observed (Supplemental Table 5).

Finally, we searched for potential correlations of EZH2 mRNA expression levels with clinical outcome and found that EZH2^high^ cases displayed significantly shorter time-to-first-treatment (TTFT) and shorter overall survival (OS) on univariate analysis (Supplemental Figure 7A, B). However, high EZH2 expression did not retain independent significance in the multivariate analysis probably due to its strong association with U-CLL (35/48 or 73% of the EZH2^high^ expressing cases concerned U-CLL). In order to explore potential TTFT and OS differences in cases with similar IGHV SHM status but different EZH2 expression levels, we analysed separately U-CLL and M-CLL cases, which we subdivided into EZH2^high^cases and EZH2^low^ subgroups. Within U-CLL, EZH2^high^ cases showed a trend for shorter TTFT compared to EZH2^low^ cases, though not reaching statistical significance (EZH2^high^ median TTFT = 1.7, EZH2^low^ median TTFT = 3.3 years, *p* = 0.14), likely due to the low number of U-CLL EZH2^low^ cases (*n* = 18) (Supplementary Figure 8)

## DISCUSSION

We recently showed that EZH2 is overexpressed in stereotyped CLL subset #1, a paradigmatic aggressive stereotyped subset of CLL cases displaying a distinctive immune signalling profile. [15, 22, 24, 25] Prompted by these initial observations, we here performed a detailed molecular and functional analysis of EZH2 in a large series of CLL cases in order to better characterize its role in CLL pathophysiology.

The findings of the present study highlight EZH2 as an important novel player in the puzzle of clinical aggressiveness in CLL. EZH2 mRNA and protein expression levels were found to be significantly higher in U-CLL compared to M-CLL and, moreover, they increased with disease progression. Notably, subset #2, another aggressive subset mostly concerning M-CLL, had low EZH2 levels, similar to the remaining M-CLL cases despite having a significantly worse outcome. [26] Consequently, it can be argued that EZH2 expression is not a uniform mechanism of aggressiveness in CLL but rather that it contributes specifically to the inferior prognosis of U-CLL.

In cancer, EZH2 hyperactivity can be caused by activating *EZH2* gene mutations or overexpression due to defective regulation [27]. The former possibility seems remote in CLL, as indicated by our targeted re-sequencing study of 92 CLL cases belonging to U-CLL stereotyped subsets, including subset #1 (unpublished data); furthermore, EZH2 mutations have not so far been identified in published NGS studies in CLL. [28–30]

Regarding the possibility that overexpression might be due to defective regulation of EZH2 expression, several mechanisms may be implicated. First, the EZH2 transcript is known to be regulated by several miRNAs [27], amongst which miR-101 targets the 3′UTR of EZH2 mRNA and promotes its degradation [31]. We have previously proposed that miR-101 regulates EZH2 expression in CLL, since we showed that silencing or overexpression of miR-101 in primary CLL cells leads to overexpression and downregulation of EZH2 protein expression, respectively [15]. Here, in a significantly larger cohort, we document an inverse correlation of EZH2 expression and miR-101 in U-CLL, thus, corroborating our previous finding [15] that miR-101 is indeed implicated in the regulation of EZH2 expression. One possible explanation for this differential effect in U-CLL versus M-CLL is that other molecular mechanisms besides miR-101 may modulate EZH2 expression, perhaps superseding and/or complementing the role of miR-101, an aspect that needs further investigation. Additionally, upregulation of EZH2 may be caused by immune triggering, which is highly relevant in the broader context of microenvironmental interactions in CLL, especially for U-CLL cases that are more responsive. [25, 32] Indeed, Iannetti et al [33] showed that CLL cell stimulation with the CD40 ligand, which strongly induces the alternative NF-κB pathway, also induces EZH2 expression, leading to suppression of a subset of p53 target genes previously associated with the senescent cell phenotype, including *DEK* and *RacGAP1*. Furthermore, c-Rel was also recently found to be a critical activator of EZH2 transcription in activated primary murine B and T cells, and also in human leukemia and multiple myeloma cell lines. [34] Finally, our preliminary data indicate EZH2 upregulation in U-CLL after TLR9 stimulation (data not shown). On these grounds it can be argued that the interplay between immune signaling and microRNAs is the major mechanism underlying EZH2 overexpression in U-CLL, opening new directions for future research.

For EZH2 to be functional, it must first assemble with its partners SUZ12, EED, EZH1, RBBP4 and RBBP7 in order to form the PRC2 complex. [3] A reasonable question then is what might happen when excess EZH2 accumulates in a cancer cell: a plausible answer could be that the consequent assembly results in abnormally high levels of PRC2, which then hypermethylates H3K27, ultimately leading to the silencing of target genes. [4] In addition, the PRC2 complex can co-operate with the PRC1 complex which also leads to the silencing of gene targets, while both PRC2 and PRC1 antagonize the Trithorax complexes. [23] On these grounds and also considering that many of these epigenetic enzymes are deregulated in various types of cancers [35], we also profiled their expression using PCR arrays and found that the main PRC2 components SUZ12, EED, EZH1, RBBP4 and RBBP7 are expressed in CLL cells in a fashion concordant to that of EZH2, hence being available for assembling functional complexes with the overexpressed EZH2.

In order to obtain insight into the potential functional impact of EZH2 in CLL, we subdivided CLL cases based on the mRNA expression levels into EZH2^high^ and EZH2^low^ subgroups and found that the former displayed higher H3K27me3 levels, supporting an association between EZH2 expression and enzymatic activity. High levels of EZH2 correlated with higher viabilty of CLL cells, while EZH2 knock-down induced CLL cell apoptosis, allowing us to argue that EZH2 is implicated in the anti-apoptotic phenotype of U-CLL clones. Interestingly, we also found that EZH2 expression was significantly more frequent amongst proliferating (Ki67^+^) versus resting (Ki67^−^) cells, indicating that it is implicated in the renewal of the CLL clone, in keeping with other B-cell lymphomas where high EZH2 levels are correlated with a high proliferative index. [36–38]

Further decisive support for a role of EZH2 in the apoptosis resistance exhibited by CLL cells with high EZH2 expression was provided by our pharmacological experiments in primary CLL cells, whereby treatment with EZH2 inhibitors led to reduced H3K27me3 levels and induction of apoptosis. This finding is similar to what has previously been reported in the DLBCL cell line WSU-DLCL2, where pharmacological inhibition of EZH2 causes cell cycle arrest in the G1 phase and then cell apoptosis, accompanied by reduction of H3K27me3 levels and upregulation of EZH2 target genes. [39]

Two further findings of the present study merit comment, especially from a clinical perspective. High EZH2 expression was found to be associated with shorter TTFT and OS, although it did not retain independent significance in the multivariate analysis, likely due to cohort size or, equally plausible, the strong association with U-CLL. More interesting, however, was the finding that within U-CLL high EZH2 expression and *TP53* aberrations showed little overlap, highlighting EZH2 overexpression as a potential p53-independent pathomechanism of clinical aggressiveness.

In conclusion, EZH2 is overexpressed in CLL cases with aggressive clinical course, is associated with disease progression and gives a survival advantage to the cells. Consequently, pharmacological inhibition of EZH2 may represent a potential novel therapeutic approach for a subgroup of CLL patients, an enticing idea that is readily achievable, since EZH2 pharmacological inhibitors are currently in clinical trials for other types of cancer, including B cell malignancies.

## MATERIALS AND METHODS

### Patient group

Blood samples were collected under informed consent from 145 patients diagnosed with CLL according to the guidelines of the International Workshop Chronic Lymphocytic Leukemia/National Cancer Institute (iwCLL/NCI). [40] All patients were either untreated or off therapy for at least 6 months prior to sampling. The study was approved by the local Ethics Review Committee of the participating institutions. Demographic, clinical and biological data for the patient cohort are given in Table 1 and Supplemental Table 1.

**Table 1:** Overview of the analyzed CLL cohort

Parameter	Number
**Gender**	
Male	95
Female	50
**IGHV gene somatic hypermutation status**	
Mutated	82
Unmutated	62
ND	1
**Binet stage at diagnosis**	
A	117
B	21
C	7
**CD38 expression**^a^	
Positive	28
Negative	98
ND	19

a >30% cut-off value for positivity, ND=Non Determined

### PCR amplification and sequence analysis of IGHV-IGHD-IGHJ rearrangements

Reverse transcriptase-polymerase chain reaction (RT-PCR) of IGHV-IGHD-IGHJ rearrangements was performed as previously described [17]. Purified PCR amplicons were subjected to direct sequencing on both strands. Sequence data were analyzed using the IMGT^®^ databases [41] and the IMGT/V-QUEST tool (http://www.imgt.org) [42].

### Cell enrichment

CD19^+^ B cells were negatively selected from whole blood using the RosetteSep B-cell enrichment kit (StemCell Technologies, Vancouver, BC, Canada) as previously described. [24] The purity of all preparations was checked by flow cytometry and always exceeded 95% for CD19^+^ cells.

### Quantification of EZH2 mRNA expression

Total cellular RNA was isolated from purified B cells with the Qiagen RNAeasy mini kit (QIAGEN, Hilden, Germany), including a DNAse incubation step. Quantification of EZH2 mRNA levels was achieved by RQ-PCR, using the specific RT^2^ qPCR Primer Assay for the EZH2 human gene (QIAGEN, Hilden, Germany), according to the manufacturer's instructions. Briefly, one microgram of RNA was reversed transcribed to cDNA and a 1:50 aliquot of the RT product was used as the template for RQ-PCR. The *ABL* gene was used as reference (housekeeping gene). For RQ-PCR experiments all samples were run in triplicate. Data were analyzed using the 2^−ΔΔCt^ algorithm. [43]

### Gene expression profiling

Gene expression profiling of 86 genes (Supplemental Table 4) belonging to the Polycomb and Trithorax complexes was performed by real-time quantitative PCR on PCR arrays using the RT^2^ Profiler™ PCR Array kit (PAHS-506Z, Qiagen) according to the manufacturer's instructions. The commercial array was modified in order to also include SUZ12, a component of the PRC2 complex, and WHSC1, also known as MMSET, which binds to transcriptionally active regions of the genome [44] and specifically catalyzes H3K36 dimethylation, a mark associated with regions of open chromatin; of note, MMSET expression has been tightly correlated to that of EZH2 in various cancer types. [42] Data analysis was performed as previously described. [24]

### Western blotting

Total cellular protein was isolated from purified B cells, run on 10% NuPAGE Bis-Tris gel (Invitrogen, Paisley, UK) and transferred to PVDF membranes (Invitrogen, Paisley, UK) as previously reported. [25] The antibodies for EZH2 and H3K27me3 were from BD Biosciences and Millipore, respectively. To ensure equal loading, membranes were stripped and reprobed with anti-actin and anti-histone H3 antibodies (Sigma-Aldrich, Taufkirchen, Germany and AbCam, respectively). Immunoreactivity was revealed by incubation with goat anti-mouse or rabbit Ig (ThermoFisher Scientific Waltham, MA USA) conjugated with horseradish peroxidase and followed by enhanced chemiluminescence reaction (Pierce, Rockford, IL). Ratios of each protein band intensity relative to the respective housekeeping gene band intensity were calculated for each sample using the ImageJ software.

### Cell culture and analysis of apoptosis in CLL cells by flow cytometry

Purified CD19^+^ B cells (3×10^6^ cells/ml) were cultured in RPMI 1640 medium supplemented with 10% fetal calf serum, 2 mmol/l l-glutamine and 15 μg/ml gentamicin (Sigma-Aldrich, Taufkirchen, Germany) in 24-well plates. Cell cultures were maintained at 37°C in a humidified atmosphere containing 5% CO_2_ for 9 days. Cells were collected at time 0 and after 9 days, washed twice and stained for AnnexinV and Propidium Iodide (PI) using the BD FITC Annexin V Apoptosis Detection Kit I, or BD Horizon Fixable Viability Staining (FVS) (BD Bioscience, San Jose, CA) to measure cell viability. Data acquisition followed on a BD FACSCalibur flow cytometer (BD Bioscience, San Jose, CA).

### Determination of Ki67 and EZH2 expression by flow cytometry

Intracellular staining for Ki67 and EZH2 expression was performed using the BD Cytofix/Cytoperm kit. In brief, cells were washed with PBS and stained with BD Horizon Fixable viability stain 660 (BD Biosciences) for 10min. The excess stain was washed off with PBS containing 1% FBS and cells were fixed/permeabilized, with Fix/Perm buffer for 20 minutes prior to the addition of the conjugated antibodies. Antibodies used were FITC Mouse Anti-Human Ki-67 (BD Biosciences), PE Mouse anti-EZH2 (BD Biosciences), CD5-PC5 (Beckman Coulter), FITC Mouse Isotypic control (BD Biosciences) and IgG1-PE isotypic control (Beckman Coulter). Data acquisition followed on a BD FACSCalibur flow cytometer (BD Bioscience, San Jose, CA). Data analysis was performed using the Flowing Software Version 2.5.

### Transfection of CLL cells with siRNA against EZH2

Purified CD19^+^ B cells from 3 CLL cases with high EZH2 mRNA levels as determined by RQ-PCR were transfected with siRNA against human EZH2, using the Amaxa Human B Cell Nucleofector Kit (Lonza, Colonge, Germany). In detail, 5×10^6^ CD19^+^ B cells from each case were resuspended in 100 μl of Human B Cell Nucleofector Solution and mixed with 70nM of 5 FlexiTube siRNAs with different sequences directed against human EZH2 (Qiagen, Hilden, Germany). In parallel, 5×10^6^ CD19^+^ B cells of each patient were transfected with 40nM of AllStars Negative Control siRNA (Qiagen, Hilden, Germany). Transfections were performed using the Amaxa Nucleofector device with the U-15 program (Lonza, Colonge, Germany). Transfection efficiency was measured with the Block-iT Alexa Fluor Red Fluorescent Oligo (Invitrogen, Paisley, UK). CLL cells were collected and processed for immunoblotting and apoptosis analysis at indicated time points.

### Construction of CRISPR lentiviral construct pLENTICRISPRv2-EZH2g.RNA

The lentiCRISPRv2 lentiviral vector obtained from Addgene (#52961) [45] was digested with *Bsm*BI to remove a filler DNA fragment. A guide RNA sequence targeting EZH2 transcript variant 1 (GenBank #NM_004456) was integrated into the linearized lentiviral vector by ligation of a double-stranded oligonucleotide formed by EZH2-gRNA-F 5′-caccgagaagggaccag tttgttgg-3′ and EZH2-gRNA-R 5′-aaacccaacaaactggtcccttctc-3′ complementary oligonucleotide sequences which were previously annealed. Proper integration of the target sequence was confirmed by Sanger sequencing using a primer from the U6 promoter region, U6 Seq F 5′-actatcatat gcttaccgtaac-3′.

The lentiviral vector was packaged by transfecting HEK293T cells (app. 6×10^6^) with 3μg plentiCRISPRv2-EZH2g.RNA, 1μg pVSV-G and 2μg pΔ8.91 plasmids by lipid-mediated delivery. The packaged virus was collected after 48 hours from supernatant filtered through a 0.45μm syringe filter. A parallel control transduction was carried out using a pLenti/K-dCas9 expressing an inactive form of Cas9. The transfection was monitored by Western blot against the FLAG fusion of the Cas9 protein. To transduce MEC-1 cells, approximately 1 million cells were pelleted and resuspended in 1ml of the filtered supernatant. 1μl of a 10μg/ml polybrene solution was added and mixed. On day 2, the transduction solution was replaced with normal growth medium RPMI complete + 10% FBS + 15 μg/ml gentamycin and on day 3 it was replaced with growth medium supplemented with 0.5μg/ml puromycin. Cells selected for >16 days were tested for Cas9 expression by Western blot with an anti-HA antibody (Cell Signaling). Deletions in the EZH2 target sequence were verified by PCR using EZH2 flanking primers EZH2-V1-F 5′-ataaaagcgatggcgat tgggct-3′ and EZH2-V1-R 5′-tctgattttacacgcttccgcca-3′.

### Inhibition of H3K27me3 trimethylation using EZH2 pharmacological inhibitors

Purified CD19^+^ B cells (3×10^6^ cells/ml) were cultured as described above in the presence of EZH2 inhibitors GSK343 (Glaxo SmithKline, UK) and EPZ6438 (Epizyme, Cambridge, MA). In more detail, cells were treated with DMSO or increasing concentrations of GSK343 or EPZ6438 for 6 days. Cells were then collected, washed and lysed for immunobloting. For selected cases, cells were treated with 4uM GSK343 and 3uM EPZ6348 for a period of 13 days. At indicated time points, cells were collected and processed for immunobloting (days 0, 2, 4, 6, 8 and 10) and apoptosis analysis (days 0, 5, 7, 9 and 13).

### Statistical analysis

Descriptive statistics for categorical variables included frequency distributions while for quantitative variables, statistical measures included mean, median and standard deviation. Associations between categorical variables were evaluated using the x^2^-test. Significance of bivariate relationships between factors and variables normally distributed was assessed with the use of Student's t-test. In cases where the underlying distribution was not normal, the Mann-Whitney test was performed. Correlation analysis was performed using the Spearman correlation coefficient.

Classification of CLL cases into EZH2^high^ and EZH2^low^ subgroups based on EZH2 mRNA relative expression (2^-ΔCt) was determined by ROC curve analysis. U-CLL cases were used as control group and the cut-off value was chosen in order to ensure high specificity and sensitivity. This threshold was set at 1.087 and confirmed by applying the Youden Index test. This threshold led to U-CLL cases being assigned into the EZH2^low^ group, and M-CLL cases into the EZH2^high^ group, respectively. For all comparisons, a significance level of *p* < 0.05 was set.

Regarding survival analysis, time-to-first-treatment (TTFT) was evaluated from the diagnostic date until the date of initial treatment (untreated cases were censored at the time of their last follow-up) while, overall survival (OS) was measured from the date of diagnosis until the last follow-up or death. Survival curves were constructed using the Kaplan-Meier method, and the log-rank test determined differences between survival proportions. Multivariate Cox regression models were used to test the simultaneous effect of factors on outcomes taking into account the relative effect of remaining parameters. All statistical analyses were performed with the use of the GraphPad Prism 5 software (GraphPad Software, La Jolla, CA) and the Statistica Software 10.0 (Stat Soft Inc, Tulsa, OK).

## SUPPLEMENTARY MATERIAL FIGURES AND TABLES




