# Supplementary material for: The histone methyltransferase EZH2 as a novel prosurvival factor in clinically aggressive chronic lymphocytic leukemia
Source: Oncotarget. 2016 May 14;7(24):35946–59. doi: 10.18632/oncotarget.9371 (PMC5094974; doi:10.18632/oncotarget.9371)
Supplement: Supplementary file 1 [file oncotarget-07-35946-s001.pdf]

The histone methyltransferase EZH2 as a novel prosurvival factor in clinically aggressive chronic lymphocytic leukemia

Supplementary Material

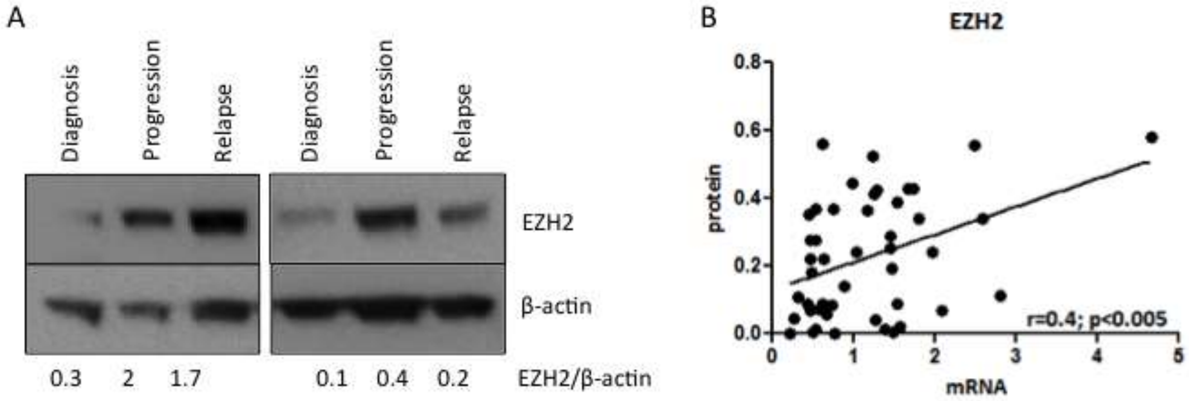

Supplemental Figure 1. (A) Western blotting analysis for EZH2 protein expression in serial samples obtained from 2 progressive U-CLL cases. The samples were obtained at diagnosis, at progression and at relapse. (B) Correlation of EZH2 mRNA and protein levels. The x axis depicts EZH2 relative expression, while the y axis depicts EZH2 protein levels normalized to β-actin.

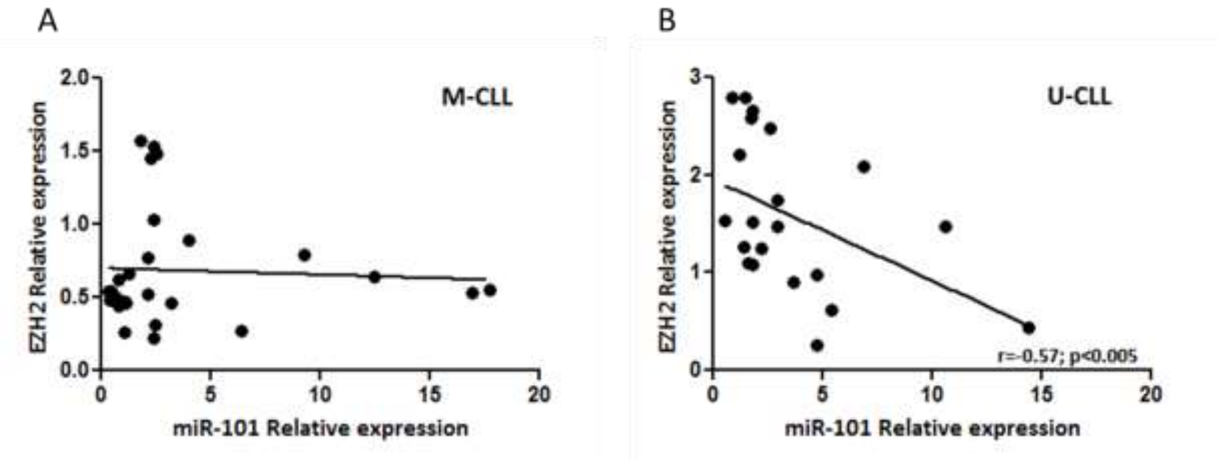

Supplemental Figure 2: miR-101 regulates EZH2 expression in U-CLL. A) EZH2 mRNA levels are not correlated with miR-101 levels in M-CLL but (B) are significantly anti-correlated (r=-0.6, p<0.005) with miR-101 levels in U-CLL.

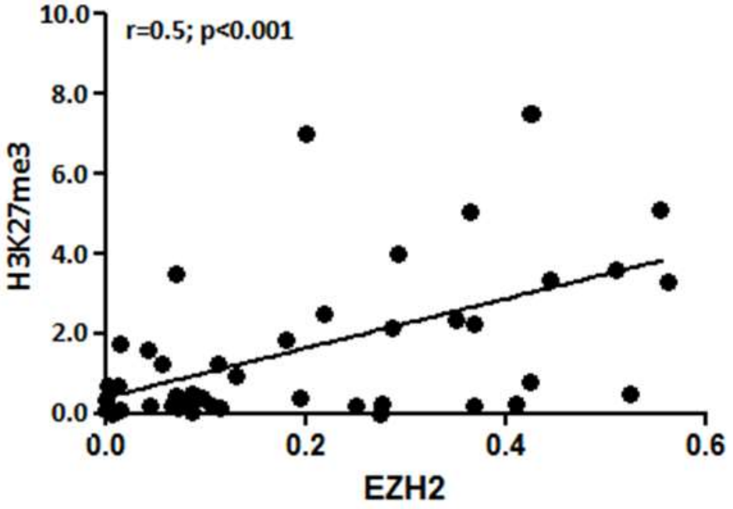

Supplemental Figure 3: Spearman correlation coefficient analysis shows that EZH2 protein levels and H3K27me3 levels are linearly correlated (r=0.5, p<0.001).

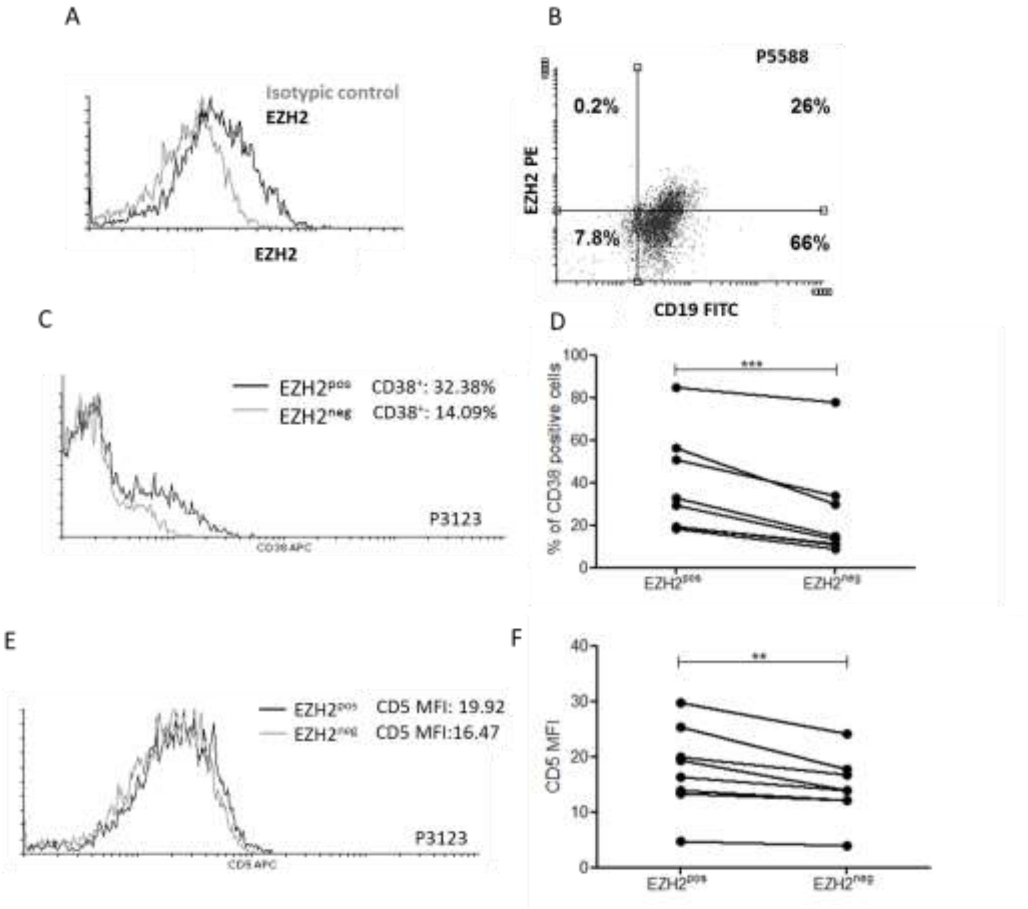

Supplemental Figure 4. A, B) EZH2 protein is expressed in a fraction of the CLL clone. (A) Single parameter histogram showing EZH2 expression for one representative case. (B) Dot plot representing CLL cells stained using FITC-labeled CD19 antibody and PE-labeled EZH2 antibody for one representative case. (C-F) Expression of additional phenotypic features in EZH2 positive versus negative fractions. Percentage of cells expressing CD38 in the two fractions in (C) one representative case (histogram) and (D) all cases analyzed (two connected points in the graph represents CD38 expression in the two different fractions for one case). Mean fluorescence intense (MFI) of CD5 in the two fractions in (E) one representative case (histogram) and (F) all cases analyzed (two connected points in the graph represents CD5 MFI in the two different fractions for one case). $^{**}p<0.05$, $^{***}p<0.0005$

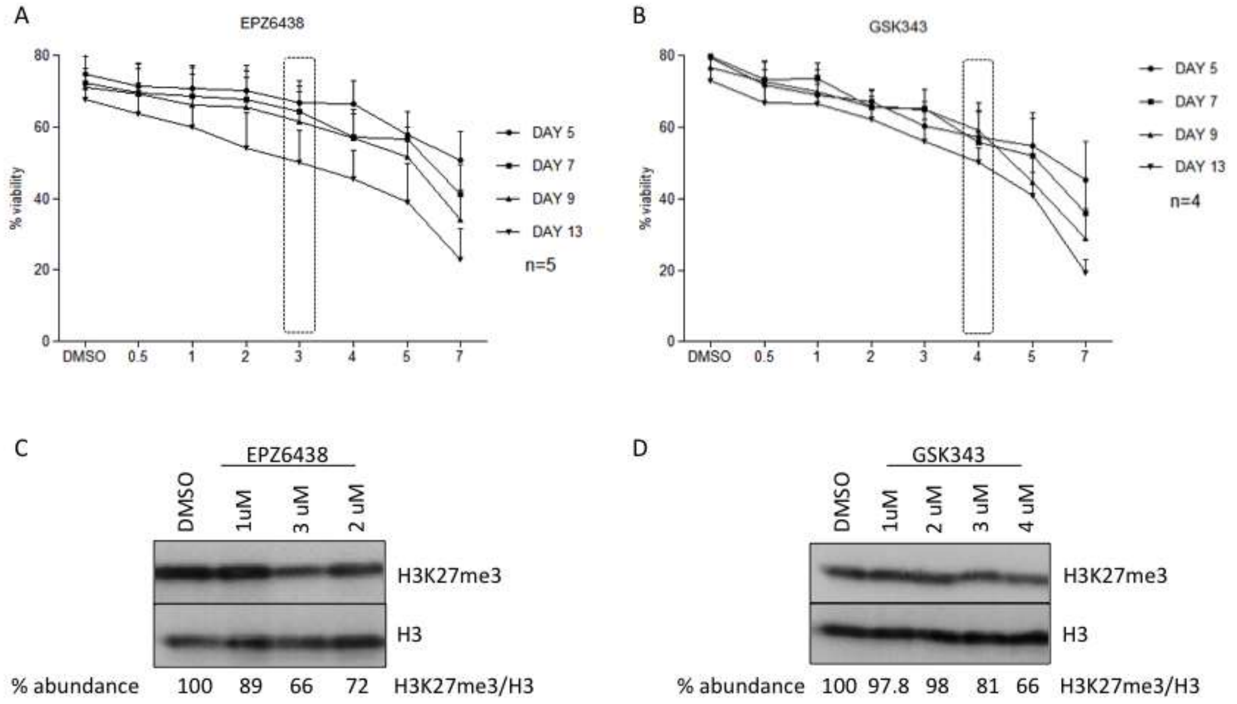

Supplemental Figure 5. Mean percentage of cell viability of CLL cells from 5 cases treated with different concentrations of both (A) EPZ6438 and (B) GSK343 inhibitors, for different time points. Western blotting for H3K27me3 levels after inhibition with different concentrations of (C) EPZ6438 and (D) GSK343 for one representative case.

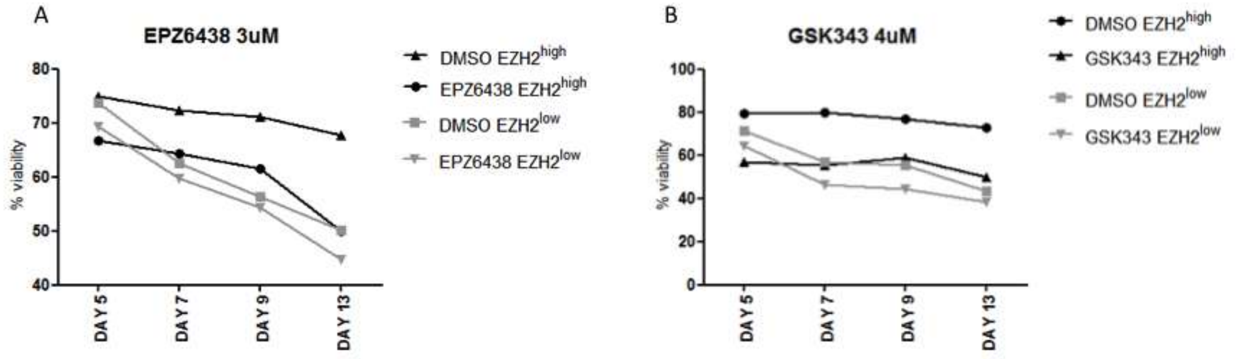

Supplemental Figure 6. Mean percentage of viable cells in 5 EZH2^{high} cases (black lines) and 3 EZH2^{low} cases (grey lines). (A) The cells were treated either with DMSO as control, or with 3uM EPZ6438. (B) The cells were treated either with DMSO as control, or with 4uM GSK343

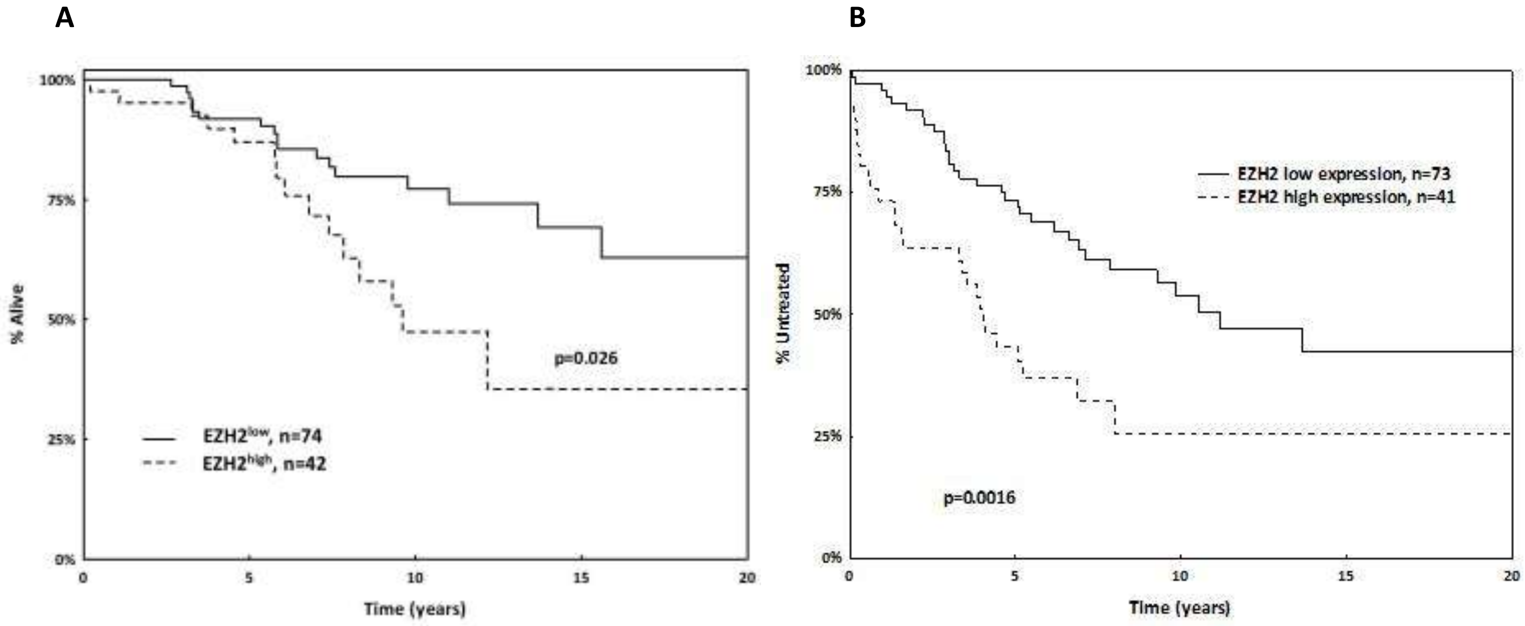

Supplemental Figure 7: Kaplan Meier curves for (A) time-to-first-treatment (TTFT) and (B) Overall survival (OS). $EZH2^{high}$ cases exhibited shorter TTFT and OS ($p<0.05$) compared to $EZH2^{low}$ cases (median TTFT and median OS: 4 and 9.6 years for $EZH2^{high}$ cases vs 11.2 and not yet reached for $EZH2^{low}$ cases, p=0.0016 and p=0.026 respectively).

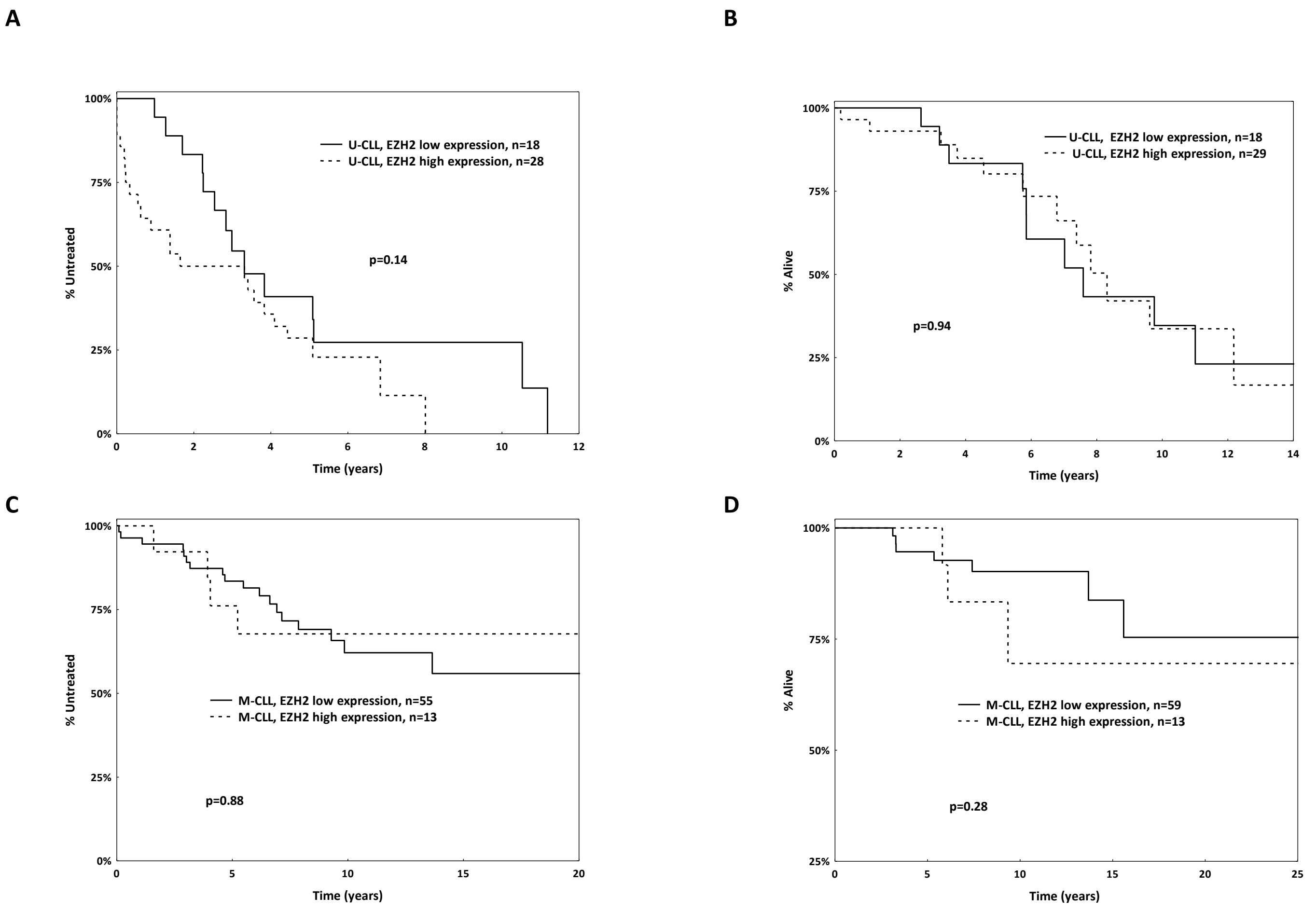

Supplemental Figure 8: Kaplan Meier curves for (A) time-to-first-treatment (TTFT) for U-CLL EZH2^{high} and EZH2^{low} cases, (B) Overall survival (OS) U-CLL EZH2^{high} and EZH2^{low} cases, (C) TTFT for M-CLL EZH2^{high} and EZH2^{low} cases and (D) OS for M-CLL EZH2^{high} and EZH2^{low} cases. U-CLL/EZH2^{high} cases show a trend for shorter TTFT compared to U-CLL/EZH2^{low} cases, though not reaching statistical significance (EZH2^{high} median TTFT= 1.7 EZH2^{low} median TTFT =3.3 years, p=0.14).

Supplemental Tables

Table S1: Demographic, clinical and biological data for the patient cohort

For Table S1, please see the attached Excel file

Supplemental Table 2: Relative expression of EZH2 and comparisons between different immunogenetic subgroups of the disease, including stereotyped subsets.

Subgroup	Relative Expression	Comparison	FD	p value
M-CLL	0.60	U-CLL vs M-CLL	2.04	0.0000
U-CLL	1.21	#1 vs #4	2.24	0.0127
#6 (U-CLL)	1.28	#6 vs #4	2.44	0.0072
#8 (U-CLL)	1.19	#8 vs #4	2.27	0.0188
#1 (U-CLL)	1.18	#1 vs #2	1.81	0.0342
#4 (M-CLL)	0.53	#6 vs #2	1.97	0.0184
#2 (mixed SHM status, mostly M-CLL)	0.65	#8 vs #2	1.83	0.0412

U-CLL: CLL cases with unmutated IGHV genes; M-CLL: CLL cases with mutated IGHV genes; #1: stereotyped subset #1; #2: stereotyped subset #2; #4: stereotyped subset #4; #6: stereotyped subset #6; #8: stereotyped subset #8; FD: Fold Difference

Supplemental Table 3: Genetic changes in 6 progressive U-CLL cases analysed for EZH2 expression on serial samples

A. Classic cytogenetic results

PATIENT ID	DIAGNOSIS	PROGRESSION TO 1st TREATMENT	RELAPSE	DIAGNOSIS	PROGRESSION TO 1st TREATMENT	RELAPSE
P4656	08/11/06	04/08/08	09/10/13	46, XY, del(6)(q21)[1]/ 46, XY[25]	46, XY, del(6)(q21)[8]/ 46,XY[12]	ND
P5283	18/05/07	23/02/10	16/05/12	46, XY, inv(9)(p11;q13)[20]	ND	46,XY,inv(9)(p12q13),add(16)(q12),der?(17)[5]/46,XY,inv(9)(p12q13)[37]
P2355	18/11/04	10/11/09	18/02/10	46,XY[20]	ND	44,XY,add(12)(p11)[4],dic(12;22)(p11.2;p11.1)[12],dic(14;18)(p11;p11)[2] dic(17;18)(p12;p11.1)[14],+mar1[2][cp16] / 46,XY[7]
P7250	05/08/08	03/05/10	05/06/12	46,XY[20]	46,XY,del(1)(q32q42)[11] /46,XY[11]	46,XY,del(1)(q32q42)[6]/46,sl,add(7)(p22)[4]/46,XY, add(15)(q22)inc[2]/46,XY[12]
P14197	12/03/09	16/04/13	27/05/14	ND	46,XY,del(13)(q12q14)[2] /46,XY[18]	46,XY,del(13)(q12q14)[10]/46,sl,t(3;17)(p21;p1?.3)[2]/46,XY[18]
P11323	30/05/11	01/05/13	20/01/14	46,XY[20]	46,XY,del(1)(q32q42)[6]/ 46,sl,add(7)(p22)[4]/ 46,XY,add(15)(q22)inc[2] /46,XY[12]	ND

ND: not determined

Supplemental Table 3B. FISH results

				Diagnosis				Progression to 1st treatment				Relapse			
**Patient ID**	**Diagnosis**	**Progression to 1st treatment**	**Relapse**	**13q-**	**+ 12**	**11q-**	**17p-**	**13q-**	**+ 12**	**11q-**	**17p-**	**13q-**	**+ 12**	**11q-**	**17p-**
P4656	08/11/06	04/08/08	09/10/13		N	N	N	N	N	N	N				
P5283	18/05/07	23/02/10	16/05/12												
P2355	18/11/04	10/11/09	18/02/10	P		N	N					P	N	N	P
P7250	05/08/08	03/05/10	05/06/12	N	N	N	N	P	N	N	N	P	N	N	N
P14197	12/03/09	16/04/13	27/05/14					P	N	N	N	P	N	N	P
P11323	30/05/11	01/05/13	20/01/14	N	N	N	N	N	N	N	N				

Empty cells denote no available data. N: negative; P: positive.

Supplemental Table 3C. *TP53* mutation screening

Patient ID	Diagnosis	Progression	Relapse	DIAGNOSIS	PROGRESSION	RELAPSE
P4656	08/11/06	04/08/08	09/10/13		N	N
P5283	18/05/07	23/02/10	16/05/12			
P2355	18/11/04	10/11/09	18/02/10	N		
P7250	05/08/08	03/05/10	05/06/12	N		
P14197	12/03/09	16/04/13	27/05/14			N
P11323	30/05/11	01/05/13	20/01/14	N	N	N

Empty cells denote no available data. N: negative

Table S4: The 86 genes of the Polycomb and Trithorax complex components that were analyzed by real-time quantitative PCR on PCR arrays. Genes with fold difference values >1 are over-expressed in U-CLL compared to M-CLL cases, while genes with fold difference values <1 are over-expressed in M-CLL compared to U-CLL cases

For Table S4, please see the attached Excel file

Supplemental Table 5: Main clinicobiological features in $EZH2^{high}$ versus $EZH2^{low}$ expressing cases.

	EZH2 high (n=48)	EZH2 low (n=83)	p value
**U-CLL**	35/48 (73%)	22/83 (27%)	**<0.0001**
**Male**	32/47 (68%)	46/77 (60%)	0.35
**<55 years**	13/42 (31%)	23/74 (31%)	0.97
**>71 years**	9/42 (21%)	13/74 (18%)	0.61
**Median age**	63 years	62 years	ns
**Binet A**	28/42 (67%)	66/74 (89%)	**0.003**
**Binet B**	11/42 (26%)	8/74 (11%)	**0.03**
**Binet C**	3/42 (7%)	0/74 (0%)	**0.02**
**Binet B+C**	14/42 (33%)	8/74 (11%)	**0.003**
**MD**	36/41 (88%)	52/68 (76%)	0.14
**Kappa**	30/42 (71%)	49/73 (67%)	0.63
**CD38**[a]	11/41 (27%)	12/71 (17%)	0.21
**Del(17p)**[b]	1/37 (3%)	2/52 (4%)	0.76
**Del(11q)**[c]	7/38 (18%)	4/54 (7%)	0.11
**Trisomy 12**[d]	5/33 (15%)	10/50 (20%)	0.57
**TP53 mut**	2/28 (7%)	4/23 (17%)	0.25
**TP53abn**	2/41 (5%)	6/61 (10%)	0.36
**del(13q)**[e]	11/33 (33%)	16/50 (32%)	0.89
**Normal FISH**	14/33 (42%)	22/50 (44%)	0.88
**NOTCH1**	1/34 (3%)	2/68 (3%)	0.99

a: >30% cut-off value for positivity, b: >2.5; c: >3.3; d: >2.65; e: >5.1
